# Research on provincial-level medical insurance coordination in Guangxi’s multi-ethnic regions from the perspective of urban–rural integration

**DOI:** 10.3389/fpubh.2026.1796550

**Published:** 2026-05-22

**Authors:** Yang Zhao, Hongji Qin, Tingting Li, Tao Jiang, Shuangling Qin

**Affiliations:** 1 School of Humanities and Management, Guilin Medical University, Guilin, China; 2 School of Clinical Medicine, Guilin Medical University, Guilin, China; 3 School of Public Health, Guilin Medical University, Guilin, China; 4 School of Marxism, Guilin Medical University, Guilin, China

**Keywords:** adjustment fund mode, basic medical insurance, Guangxi province, provincial-level overall planning, unified revenue and expenditure mode

## Abstract

**Objective:**

This study aims to explore the necessity, model selection, and implementation suggestions for the provincial-level overall planning of basic medical insurance in Guangxi, China, to address the structural imbalance of medical insurance funds and improve the sustainability of the system.

**Methods:**

A comparative analysis approach was employed to examine the “unified revenue and expenditure” model and the “adjustment fund” model for provincial-level overall planning. Data were collected from official sources, including the 2023 Statistical Communique on the Development of Medical Security in Guangxi and policy documents. The analysis focused on fund balance, demographic structure, and regional disparities in Guangxi.

**Results:**

In 2023, the accumulated balance of Guangxi’s employee medical insurance pooling fund reached 37.736 billion yuan, and the resident medical insurance fund balance was 43.197 billion yuan. However, significant regional imbalances existed, with the number of months covered by the accumulated balance being 22.40 months for employee insurance and 11.51 months for resident insurance. Cross-regional medical treatments reached 5.888 million visits, costing 19.165 billion yuan, leading to a “siphoning effect” (i.e., the excessive concentration of patients and funds towards medical resource-rich areas) that exacerbated regional disparities. The adjustment fund model is recommended as an initial step for Guangxi, given its flexibility and lower reform resistance.

**Conclusion:**

Promoting provincial-level overall planning of basic medical insurance in Guangxi is essential to solve fund imbalances and improve sustainability. The adjustment fund model serves as a transitional approach, with gradual movement toward unified revenue and expenditure. Key suggestions include establishing dual-track adjustment funds, scientific fund extraction rules, and enhancing digital infrastructure.

## 1 Introduction

### 1.1 Theoretical foundations and literature review

The theoretical basis for elevating the level of medical insurance pooling primarily stems from two academic traditions: the law of large numbers in insurance actuarial science and fiscal federalism theory in public finance. This section will systematically review relevant domestic and international research to provide theoretical support for analyzing the issue of provincial-level pooling in Guangxi.

1. The law of large numbers and risk pooling theory

The Law of Large Numbers forms the cornerstone of insurance operations. This principle states that as the number of risk units increases, actual loss outcomes increasingly converge toward expected losses, thereby reducing risk volatility. In the field of health insurance, this principle manifests as the expansion of risk pool size enhancing the fund’s risk diversification capacity. International academia has conducted in-depth discussions on risk pool design. Mathauer et al. (1)proposed an eight-category classification framework for health insurance risk pools, categorizing global practices into types such as single pools, regional multi-pools, and equalization pools. This typology provides a tool for analyzing the advantages and disadvantages of different pooling models. Flourence et al. (2) conducted a systematic review of national health insurance program experiences across 11 low- and middle-income countries (7 in sub-Saharan Africa, 4 in Asia). Their findings indicate that merging risk pools is a core strategy for advancing universal health coverage. This offers significant insights for understanding Guangxi’s transition from 15 municipal pools to a provincial adjustment pool. Domestic scholars have also researched this topic. Xu Chuangzhou (3)analyzing the New Rural Cooperative Medical Scheme, noted that the theoretical foundation of “cooperation” lies in collectively addressing uncertain medical risks through the law of large numbers. Dong (4) empirical research found that raising the coordination level significantly reduced participants’ medical burdens, with the provincial pooling model demonstrating greater health improvement potential than the adjustment fund model. Hu et al. (5) confirmed through their Gansu study that elevating coordination levels enhances the healthcare system’s resilience against sudden shocks. Mathauer et al. (1) further proposed an eight-category classification framework for healthcare risk pools, categorizing global practices into single pools, regional multi-pools, redistribution pools, and others, providing a typological tool for analyzing the advantages and disadvantages of different coordination models.

1. Fiscal federalism and the theory of jurisdictional division

Fiscal federalism theory focuses on the functional division of labor among different levels of government in the provision of public goods. In the healthcare insurance sector, this theory manifests as the allocation of healthcare insurance responsibilities and expenditure obligations among provincial, municipal, and county-level governments. International experience provides valuable reference points for understanding this issue. Salari and Crivelli (6) analyzed the equity of health insurance financing across Swiss cantons, revealing significant disparities in funding between cantons within federal systems. This necessitates balancing mechanisms at the federal level. Yan (7) recent research reveals that healthcare integration policies generate a “spatial siphon effect”—provinces with lower per capita benefits tend to cluster with neighboring provinces offering higher per capita benefits. This provides a theoretical perspective for understanding the interaction of healthcare funds across provinces. Tian et al. (8) demonstrated that the direct settlement policy for cross-provincial medical treatment significantly improves the health of rural–urban migrant populations, though this effect depends on higher-level coordination arrangements. Domestic scholars have also explored this issue from a fiscal decentralization perspective. Zhu Qinlei (9) analyzed the division of responsibilities and expenditure obligations for basic medical insurance from a fiscal federalism perspective, noting that elevating the coordination level inevitably involves restructuring intergovernmental authority and responsibility relationships. Zhang et al. (10) found significant disparities in the effects of medical insurance across different groups—rural migrant workers benefited from inpatient reimbursement but saw no improvement in outpatient out-of-pocket expenditures. This suggests that elevating the coordination level requires complementary capacity building at the grassroots service level.

1. Research review and gaps

The above studies demonstrate that the international academic community has conducted in-depth discussions on the design of health insurance risk pools, fiscal decentralization, and health insurance governance. Domestic scholars have also begun to focus on the effects and influencing factors of elevating the level of coordination. The studies by Flourence et al. (2) on low- and middle-income countries, Mathauer et al. (1)‘s risk pool classification framework, and the empirical analyses of China’s health insurance policies by Yan (7) and Tian et al. (8) provide a solid theoretical foundation and methodological reference for this paper. However, existing research predominantly focuses on the national level or developed eastern regions. Quantitative studies on coordination pathways in western multi-ethnic provinces, particularly under the constraints of urban–rural integration, remain largely unexplored. Guangxi, as a multi-ethnic autonomous region, is home to 11 ethnic minorities including the Zhuang, Yao, Miao, and Dong with ethnic minorities constituting 38% of its population (11). The operational characteristics of medical insurance funds in its 11 ethnic autonomous counties have not received sufficient attention. This constitutes the marginal contribution of this study: building upon existing theoretical frameworks, it explores provincial coordination pathways suitable for western multi-ethnic regions by integrating Guangxi’s distinctive features of multi-ethnicity and pronounced urban–rural disparities.

### 1.2 Policy context and research questions

For a long time, improving the overall planning level has always been a key measure to enhance the mutual assistance nature of social insurance and the risk resistance capacity of funds, and it is also a key issue in the construction of China’s social insurance system (12). This pursuit of higher-level coordination is driven by the need to address structural imbalances within insurance funds, enhance risk pooling across broader populations, and ultimately achieve greater systemic sustainability and equity. The move towards provincial-level planning represents a significant evolution in the management of China’s medical security system, aiming to create a more unified and efficient framework.

The policy impetus for this reform is unequivocal and stems from the highest levels of governance. The 20th National Congress of the Communist Party of China proposed to “promote the provincial-level overall planning of basic medical insurance” (13), and the Third Plenary Session of the 20th Central Committee of the Communist Party of China included it in the deepening reform plan again (14). This top-level design has been further reinforced by legislative action. Article 20 of the “Draft Law on Medical Security of the People’s Republic of China” further clearly states in the form of legislation that “the state promotes the provincial-level overall planning of basic medical insurance” (15). This creates a powerful policy-legal framework guiding the reform nationwide.

Substantial progress has been made across China in implementing this directive. As of December 2024, 16 provinces had taken the lead in completing the provincial-level overall planning of basic medical insurance. The approaches and timelines, however, have varied. Some provinces, such as Beijing, Tianjin, Shanghai, Chongqing, Tibet, Ningxia, Hainan, and Qinghai, had implemented the provincial-level overall planning of medical insurance before the national requirement was clearly defined in 2020. Shandong, Jiangxi, Shanxi, and Sichuan achieved the centralized implementation of provincial-level overall planning in 2024. Liaoning and Anijo adopted the model of “policy improvement” followed by “step-by-step promotion” (16). This demonstrates a range of viable pathways to achieving the provincial coordination goal.

However, in sharp contrast to the aforementioned provinces, Guangxi still maintains 15 isolated municipal-level overall planning regions (14 prefecture-level cities plus the regional-level directly under the autonomous region), with the fund showing a structural imbalance characterized by “idleness on one end and depletion on the other” (17). This fragmented model has resulted in significant disparities, where some municipal pools accumulate large surpluses while others face persistent deficits, undermining the system’s overall efficiency and equity. The persistence of this municipal-level planning model poses challenges to risk pooling and equitable resource distribution across the province.

Against this backdrop, this study focuses on two core questions:

What structural imbalances characterize the operation of medical insurance funds in Guangxi’s multi-ethnic regions? How significant are the disparities among different cities?

In the context of urban–rural integration, which provincial pooling model is more suitable for Guangxi? How can the “adjustment fund model” achieve “Pareto improvement” during the transition period?

Therefore, aligning with the central government’s arrangements and legislative requirements is imperative for Guangxi. Accelerating the advancement of provincial-level overall planning is crucial to ensure that insured residents across the region can equitably enjoy more reliable and sustainable medical security. This paper will explore the necessity and feasible pathways for Guangxi to transition from its current fragmented system to an integrated provincial-level coordination model, learning from the experiences of other provinces while adapting to local conditions. After stating the two core research questions, add a new paragraph: “This study not only aims to provide a pathway reference for Guangxi’s provincial-level pooling but also expects its findings—particularly the applicability analysis of the ‘adjustment fund’ as a transitional model in contexts characterized by multi-ethnicity, significant urban–rural disparities, and structural imbalances in medical insurance funds—to offer transferable experiences and a theoretical framework for other western multi-ethnic provinces in China (e.g., Yunnan, Guizhou) advancing similar reforms.

## 2 Methods

This study employs a mixed-methods design comprising three interrelated modules: First, descriptive statistical analysis based on official data aims to characterize the structural features of Guangxi’s medical insurance fund operations. Second, comparative case analysis provides insights for Guangxi’s model selection through an in-depth examination of Hainan’s provincial pooling evolution. Third, policy analysis proposes a suitable pooling pathway for Guangxi based on current status diagnosis and experiential references. It should be clarified that the Hainan case analysis is positioned as “model reference” rather than “data comparison”—focusing on examining its institutional evolution logic (transitioning from a reserve fund model to a unified collection and expenditure model) rather than conducting quantitative comparisons across years. This methodological design adheres to the principle of typicality in case studies. This study employs a mixed-methods approach to analyze the provincial-level coordination of basic medical insurance in Guangxi. The methods are structured into four key components:

### 2.1 Data collection and preprocessing

Data were collected from official sources, including the Guangxi Medical Security Bureau Statistical Communiqués (2023) and regional economic and social development reports. The dataset encompassed fund income, expenditure, accumulated balances, and demographic indicators for 14 municipal-level planning areas in Guangxi. Data preprocessing involved cleaning, normalization, and categorization to ensure consistency for comparative analysis. Tables and figures were generated to illustrate fund balances and coverage months, with reference to standards from the Ministry of Human Resources and Social Security and the Ministry of Finance. To quantify regional disparities in medical insurance funds across cities in Guangxi, this paper also compiled data on permanent residents and insured populations for each city (Table 1), which were used to calculate the weighting indicators required for the Taier Index. It should be noted that during data collection, cumulative surplus data for employee medical insurance was missing for some cities. Specifically, the cumulative surplus data for employee medical insurance in Wuzhou, Beihai, Yulin, and Baise (as shown in Table 2) could not be obtained because the 2023 statistical bulletins for these cities did not disclose this specific indicator. In subsequent analysis of the Tiel Index, these four missing samples were excluded, and this exclusion is noted in the relevant sections.

**Table 1:** Statistical table of population and medical insurance participation in Guangxi, 2023 (Unit: 10,000 persons).

Region	Permanent population	aged 60 or over	Number of medical insurance participants	
			Employees	Residents
Nanning	894.08	129.19	165.80	578.59
Guilin	495.07	100.25	80.69	409.84
Wuzhou	283.30	47.04	37.86	278.95
Beihai	188.84	29.75	29.88	137.09
Fangchenggang	106.90	16.91	17.79	77.09
Guigang	430.88	86.16	33.53	445.26
Yvlin	582.41	91.59	50.24	552.78
Baise	350.90	60.17	42.25	353.17
Hechi	335.36	60.81	36.26	360.83

Sources: Compilation of data from the 2023 Statistical Communiqués on National Economic and Social Development of Cities in Guangxi.

**Table 2:** Differences in revenue and expenditure of basic medical insurance funds across 14 municipalities in Guangxi in 2023(unit: 100 million yuan).

Region	Fund income		Fund expenditure		Accumulate balance	
	Employees	Residents	Employees	Residents	Employees	Residents
Nanning (37)	78.73	57.94	72.72	63.39	131.56	38.30
Guilin (38)	36.10	47.90	40.79	41.42	0.28	6.48
Wuzhou (39)	18.48	29.36	14.45	30.67	—	—
Beihai (40)	7.75	5.52	5.34	5.47	—	—
Fangchenggang (41)	10.44	8.09	6.99	7.27	21.49	13.70
Guigang (42)	13.13	48.43	11.42	42.57	23.40	61.58
Yvlin (43)	25.70	57.67	22.84	56.44	—	—
Baise (44)	23.52	37.10	18.21	35.65	—	—
Hechi (45)	16.13	36.40	15.76	35.71	9.53	

Sources: Data collation of the official websites of each city. Cumulative surplus data for employee medical insurance in Wuzhou, Beihai, Yulin, and Baise is missing, as these cities did not disclose this specific indicator in their 2023 statistical bulletins. Source: Data compiled from official city websites.

### 2.2 Comparative model analysis

A comparative analysis was conducted between the “unified revenue and expenditure” model and the “adjustment fund” model. This involved reviewing literature and case studies from provinces like Hainan, Beijing, and Tibet to identify characteristics, applicability conditions, advantages, and challenges of each model. Key dimensions included fund concentration, management authority, system positioning, and treatment unification. The analysis drew on economic theories such as Pareto improvement to evaluate efficiency and equity implications.

### 2.3 Statistical analysis methods for regional differences

To measure the regional disparities in the number of employees enrolled in basic medical insurance across cities, this study employs the coefficient of variation. The calculation formula for the coefficient of variation is as follows:

CV=σ/μ

where \sigma represents the standard deviation and \mu denotes the mean. The coefficient of variation eliminates dimensional effects, reflecting the relative dispersion of data. A higher CV value indicates more pronounced regional differences.

To further investigate the causes of regional disparities in enrollment numbers, this paper constructs a cross-sectional regression model:

$$Y_{i}=\alpha +\beta ₁\cdot \mathrm{Po}p_{i}+\beta ₂\cdot \text{Agin}g_{i}+\beta ₃\cdot \text{Nort}h_{i}+\beta ₄\cdot \text{Sout}h_{i}+\varepsilon_{i}.$$

Variable Definitions:

Yᵢ: Number of employees enrolled in municipal employee medical insurance for the i-th city (in ten thousand persons).

Popᵢ: Permanent resident population for the i-th city (in ten thousand persons).

Agingᵢ: Aging rate for the i-th city (proportion of population aged 60 and above, %).

Northᵢ and Southᵢ: Dummy variables for the northern and southern regions of Guangxi, respectively (with western Guangxi as the reference group).

εᵢ: Random error term.

α: Constant term.

β₁, β₂, β₃, β₄: Regression coefficients for the respective variables.

### 2.4 Case study and empirical evidence

This study uses Hainan Province as a representative case to analyze the institutional evolution path of its provincial-level pooling system. As one of China’s earliest provinces to implement provincial pooling, Hainan’s experience transitioning from the “adjustment fund model” to the “unified collection and expenditure model” holds significant reference value for Guangxi, which similarly faces pronounced regional disparities.

It is important to emphasize that the analysis in this section is positioned as “model reference” rather than “data comparison.” Data from Hainan in 2011–2012 (Tables 3, 4) primarily illustrate the “initial conditions” at the outset of its institutional evolution—namely, that before implementing the adjustment fund model, significant fund disparities existed among its cities and counties (as shown in Table 3), Haikou and Danzhou had higher surpluses, while Dongfang City had a lower surplus. This initial condition shares “typological similarity” with Guangxi’s current situation, making Hainan’s experience a valuable reference for Guangxi. This paper does not conduct direct quantitative comparisons between Guangxi’s 2023 data and Hainan’s 2011 data, but instead focuses on analyzing the logic of institutional evolution.

**Table 3:** Discrepancies in revenue and expenditure of basic medical insurance funds in Hainan’s provinces, cities, and counties in 2011 (unit: 10,000 yuan).

Region	Fund income			Fund expenditure			Accumulate balance		
	Total	Employees	Residents	Total	Employees	Residents	Total	Employees	Residents
Haikou	67,735	54,802	12,933	58,386	48,113	10,273	**33,656**	17,626	16,030
Sanya	43,739	31,878	11,861	27,894	19,216	8,678	48,202	38,490	9,712
Wuzhishan	3,809	3,235	574	2,472	2,136	336	4,957	4,237	720
Wenchang	10,616	8,960	1,656	8,103	7,291	812	9,386	7,603	1783
Qionghai	14,077	12,439	1,638	10,851	9,925	926	16,459	14,803	1,656
Wanning	14,812	12,853	1959	12,963	11,654	1,309	22,379	20,350	2029
Danzhou	29,235	25,747	3,488	19,739	17,331	2,408	41,063	37,044	4,019
Dongfang	9,289	7,808	1,481	7,286	6,283	1,003	7,984	6,673	1,311

Sources: Hainan 2012 Statistical Yearbook. This table is solely for illustrating the initial conditions prior to Hainan’s implementation of the adjustment fund model and is not intended for cross-year quantitative comparisons. Bold values indicate cumulative balances of medical insurance funds (unit: 10,000 yuan).

**Table 4:** Statistics of population and medical insurance in various regions of Hainan Province in 2011.

Population (10,000 persons)	Total Population (in 10,000 persons)	Total Insured Population (in 10,000 persons)	on-post staffs (in 10,000 persons)	Retirees (in 10,000 persons)	Ratio of Employed to Retired Persons (%)	Urban Residents (in 10,000 persons)
Haikou	162.39	86.53	32.95	6.95	4.74	46.62
Sanya	58.14	59.63	18.82	0.28	67.73	40.53
Wuzhishan	11.29	3.58	1.49	0.42	3.53	1.67
Wenchang	59.39	10.60	3.82	2.17	1.76	4.62
Qionghai	50.17	12.35	3.76	2.50	1.50	6.09
Wanning	61.42	12.98	3.46	2.57	1.35	6.95
Danzhou	109.19	28.32	10.59	3.99	2.65	13.73
Dongfang	44.91	10.45	2.72	1.15	2.37	6.59

Urban residents include adults, primary, middle and high school students. Sources: Hainan 2012 Statistical Yearbook. This table is provided solely to present the demographic background prior to Hainan’s implementation of the adjustment fund model.

### 2.5 Policy recommendation framework

**Figure 1:**
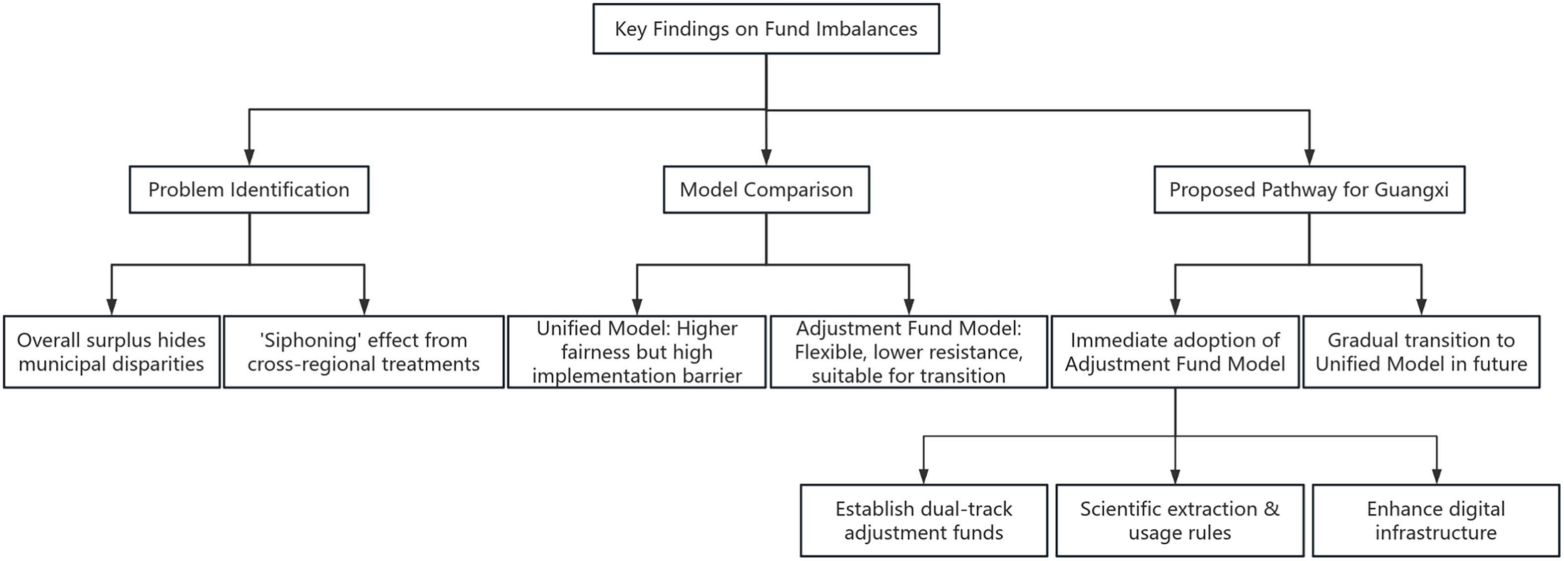
The logical diagram of the summarizing relationship of the results in the results section.

Based on the findings, a framework for policy recommendations was developed, focusing on the extraction and use of adjustment funds, incentive mechanisms, and digital infrastructure. This included designing rules for deficit adjustment, assessment adjustment, and urgent adjustment, inspired by practices from provinces like Jiangxi and Shanxi (Figure 1).

## 3 Results

### 3.1 The necessity of provincial-level overall planning for basic medical Insurance in Guangxi

#### 3.1.1 Addressing the issue of structural imbalance in medical insurance funds

According to the 2023 Statistical Communique on the Development of Medical Security in Guangxi, the accumulated balance of Guangxi’s employee medical insurance pooling fund (including maternity insurance) reached 37.736 billion yuan in 2023, and the accumulated balance of the resident medical insurance fund stood at 43.197 billion yuan (18).

In accordance with the provisions of the Guiding Opinions on Further Strengthening the Management of Basic Medical Insurance Funds (SC Doc. [2009] No. 67) jointly issued by the Ministry of Human Resources and Social Security and the Ministry of Finance, a reasonable accumulated balance of medical insurance funds should be equivalent to the average monthly payment level for 6 to 9 months. An accumulated balance exceeding the average monthly payment level for 15 months is defined as an excessive balance, while an accumulated balance lower than the average monthly payment level for 3 months is regarded as an insufficient balance (19). Calculated on this basis, the number of fund can cover payments across the region in 2023 was 22.40 months, and the number of months that the accumulated balance of the resident medical insurance fund can cover payments was 11.51 months (refer to Table 5).

**Table 5:** Cumulative surplus and coverage months of Guangxi medical insurance fund in 2023.

Type items	Pooling fund income	Pooling fund expenditure	Accumulated balance of pooling fund	Number of months covered by pooling fund balance	Reasonable number of months covered by balance	Number of months covered by excessive balance	Number of months covered by insufficient balance
Employee medical insurance	268.66	202.16	377.36	22.40 months	6–9 months	More than 15 months	Under 3 months
Resident medical insurance	475.25	450.41	431.97	11.51 months			

Data source: Data collation from Statistical Communiqué and Guiding Opinions. According to Document No. 67 [2009] issued by the Ministry of Human Resources and Social Security and the Ministry of Finance, a reasonable cumulative surplus should be equivalent to 6–9 months of average payment levels. A surplus exceeding 15 months constitutes an excess surplus, while a surplus below 3 months constitutes an insufficient surplus.

As can be seen from Table 5, although the accumulated balance of Guangxi’s resident medical insurance fund across the region reached 43.197 billion yuan in 2023, and the number of months covered by this accumulated balance was slightly higher than the upper limit of the reasonable range (9 months), in some overall planning areas, the current expenditure of the resident medical insurance fund exceeded its current income in 2023, forcing the use of accumulated balances. Medical insurance authorities are concerned that the overly rapid expenditure of the resident medical insurance fund may lead to an insufficient number of months that the remaining balance can cover.

The relatively large accumulated balance is attributed to the fact that the fund (43.197 billion yuan) is distributed across 14 overall planning areas in the region: some areas have a large surplus, while others face a balance shortage. Areas with insufficient balances have weak risk resistance capabilities for their medical insurance funds, whereas areas with excessive balances result in the idleness of medical insurance fund funds.

Similarly, the number of months covered by the accumulated balance of Guangxi’s employee medical insurance fund across the region reached 22.40 months in 2023, and there was also a phenomenon of unbalanced fund balances among the 14 overall planning areas.

According to the law of large numbers in insurance, if the overall planning level of medical insurance is elevated to the provincial level to form a unified fund pool, the adjustment and application scope of the medical insurance fund will be expanded from covering individual cities separately to covering the entire region. This will not only resolve the structural imbalance of medical insurance fund balances, characterized by both “fund depletion” and “fund idleness,” but also significantly enhance the risk resistance capacity of the fund pool and the efficiency of fund utilization.

Of particular note is that Guangxi, as a multi-ethnic autonomous region, is home to 11 ethnic minorities including the Zhuang, Yao, Miao, and Dong, with 11 ethnic autonomous counties established within its borders. The prefecture-level cities overseeing these autonomous counties include: Liuzhou (Sanjiang Dong Autonomous County, Rongshui Miao Autonomous County), Laibin (Jinxiu Yao Autonomous County), Hechi (Luocheng Mulao Autonomous County, Huanjiang Maonan Autonomous County, Bama Yao Autonomous County, Du’an Yao Autonomous County, Dahua Yao Autonomous County), Baise (Longlin Autonomous County of Various Ethnic Groups), and Guilin (Longsheng Autonomous County of Various Ethnic Groups, Gongcheng Yao Autonomous County). The operation of medical insurance funds in these prefecture-level cities differs significantly from those in prefecture-level cities with predominantly Han populations.

Data from Tables 1, 2 reveal that prefecture-level cities with a high concentration of ethnic autonomous counties (Liuzhou, Laibin, Hechi, Baise, Guilin) exhibit the following characteristics: First, the cumulative per capita fund surplus is generally below the regional average; second, fund revenues rely more heavily on fiscal transfers from higher-level governments; Third, they exhibit higher levels of population aging (e.g., Guilin’s population aged 60 and above accounts for 21.20%, exceeding the regional average of 17.45%), resulting in greater pressure on medical insurance fund expenditures. This finding underscores the need for special attention to the unique circumstances of autonomous counties in multi-ethnic regions during medical insurance pooling—cities with a concentration of autonomous counties demonstrate weaker fund resilience. When advancing provincial-level pooling, appropriate should be provided to such regions through provincial adjustment mechanisms.

It is important to note that the 2023 statistical bulletins for some cities (Wuzhou, Beihai, Yulin, Baise) did not disclose the specific indicator for “accumulated balance of the employee medical insurance pooling fund” (see Table 2). Therefore, when calculating the provincial coverage months of fund balance, we used the complete aggregate data for the entire region. However, for analyzing inter-city disparities in fund balances, these missing samples were excluded. This may lead to an underestimation of the degree of regional imbalance, as the cities with missing data are likely those with lower surpluses or less transparent fund management.

#### 3.1.2 The mechanism of provincial coordination in mitigating the “siphoning” effect of the fund

The “siphoning effect” here vividly describes the phenomenon where patients seeking cross-region healthcare cause the medical insurance funds from their insured location to flow to the treatment location, thereby exacerbating inter-regional fund imbalances.

Under the framework of 14 municipal-level fund pools operating independently in Guangxi, the comprehensive cancellation of cross-region medical treatment registration since July 2023 has directly revealed the shortcomings of the system. According to the data from the 2023 Guangxi Medical Security Development Statistical Bulletin, in 2023, the number of outpatient and emergency visits, outpatient chronic special diseases, and inpatient cross-region medical treatments reached 5.8883 million, with costs amounting to 19.165 billion yuan, compared to 3.7667 million visits and 14.729 billion yuan in 2022 (18),which marks a significant increase.

Although the original intention of the policy is to facilitate long-term stay and referral of people in other places (20), it directly triggers the concentration of “medical flow” to the medical highland (21). The point-to-point settlement mechanism of “hospital in the place of medical treatment-medical insurance bureau in the insured place” has made the funds in underdeveloped cities “lose blood” with the migration of patients, forming a reverse redistribution of “expenditure in poor cities and benefits in rich cities.” Moreover, the load on tertiary hospitals has increased sharply (22), and the phenomenon of fund “siphon” has intensified, affecting the efficiency of fund utilization.

It should be noted that mitigating the “siphoning effect” of funds is one of the anticipated positive outcomes following provincial-level pooling implementation, rather than a prerequisite for such pooling. Yan (7) reveals that medical insurance integration policies generate a “spatial siphoning effect”—provinces with lower per capita benefits tend to cluster with neighboring provinces offering higher per capita benefits. This finding provides a theoretical perspective for understanding fund imbalances caused by cross-regional healthcare seeking. Tian et al. (8) demonstrate that the direct settlement policy for cross-provincial medical treatment significantly improves the health outcomes of urban–rural migrant populations, though this effect relies on higher-level pooling arrangements. The fundamental rationale for provincial pooling lies in expanding the risk pool size to optimize fund operations through the law of large numbers, thereby enhancing the system’s mutual aid capacity and equitable sustainability. When coordination levels shift from municipal to provincial and a unified provincial risk pool is established, funds can be redistributed across the entire province. This effectively mitigates the local fund imbalances caused by the aforementioned “siphoning” effect—fund-outflow regions receive compensation through provincial redistribution, while fund-inflow regions avoid bearing excessive expenditure pressures. Consequently, academia widely recognizes alleviating interregional fund imbalances as a key policy benefit of elevating coordination levels (4).

Therefore, only through provincial coordination, the 14 decentralized funds are merged into a unified “provincial pool,” and the unified management of income and expenditure and the mutual assistance of surplus and deficiency can be cut off from the siphon channel of “medical flow determines the flow of funds” and simultaneously balance the risk of regional funds and the allocation of medical resources.

#### 3.1.3 Analysis of regional variations in employee medical insurance enrollment

Due to limitations in data availability, the cumulative surplus data for employee medical insurance funds across cities is not fully disclosed. This study employs the coefficient of variation in the number of insured individuals to indirectly reflect regional disparities. The number of insured individuals serves as a fundamental variable influencing medical insurance fund revenues and expenditures. Its regional distribution can to some extent reflect the spatial distribution of fund pressures: regions with larger insured populations generate greater fund revenues but also face increased expenditure pressures due to higher concentrations of medical resources; regions with smaller insured populations have limited fund revenues, and if simultaneously confronted with population aging, face greater challenges to fund sustainability (Table 6).

**Table 6:** Descriptive statistics of urban employee basic medical insurance participants in Guangxi by city, 2023.

Statistical indicator	Value
Total number of insured persons	6.8703 million
Mean	0.4907 million
Standard deviation	0.3864 million
Coefficient of variation	0.789
Maximum (Nanning)	1.6580 million
Minimum (Fangchenggang)	0.1779 million
Range	1.4801 million
Median	0.3353 million (Guigang)

Coefficient of Variation = Standard Deviation / Mean, reflecting the relative dispersion of data. CV > 0.50 indicates significant variation.

The coefficient of variation was 0.787, significantly higher than the 0.50 threshold for statistical significance, indicating substantial regional disparities in the number of employees enrolled in medical insurance across cities in Guangxi. Specifically:

1. Highly Concentrated Enrollment: The three cities of Nanning (1.658 million), Liuzhou (941,500), and Guilin (806,900) collectively account for 3.4064 million enrollees, representing 49.6% of the region’s total employee medical insurance enrollment. This indicates that nearly half of all enrollees are concentrated in these three central cities.
2. Significant Intra-regional Disparities: Within Northern Guangxi, Liuzhou (941,500) has 4.3 times the enrollment of Hezhou (220,600). Within southern Guangxi, Nanning (1.658 million) is 9.3 times larger than Fangchenggang (177,900); within western Guangxi, Yulin (502,400) is 2.1 times larger than Chongzuo (234,800).
3. Compounding effect with population aging: Comparing the proportion of residents aged 60 and above in Table 4, regions with higher aging rates such as Guilin (20.25%) and Hechi (18.13%) exhibit relatively fewer employees enrolled in medical insurance, further intensifying fund expenditure pressures in these areas.

#### 3.1.4 Regression analysis of factors influencing employee medical insurance enrollment

To further explain the regional disparities in employee medical insurance enrollment across Guangxi’s cities, this section constructs a cross-sectional regression model for analysis. The dependent variable is the number of employees enrolled in medical insurance in each city, while the explanatory variables include permanent resident population, aging rate (proportion of population aged 60 and above), and regional dummy variables (with Western Guangxi as the reference group). The regression results are shown in Tables 1, 7.

**Table 7:** Regression analysis results of factors influencing urban employee basic medical insurance participants.

Variable	Coefficient	Std. error	*t*-value	*p*-value
Constant	−5.62	81.13	−0.07	0.946
Permanent population	0.17***	0.03	6.48	0.000
Aging rate	−1.05	4.62	−0.23	0.826
Northern Guangxi Dummy	21.41	14.20	1.51	0.166
Southern Guangxi Dummy	17.58	14.59	1.21	0.259

***p < 0.01, **p < 0.05, *p < 0.1. Dependent variable is the number of urban employee basic medical insurance participants (in 10,000 persons). *R*^2^ = 0.842, Adjusted *R*^2^ = 0.772, *F* = 12.03 (*p* = 0.001). The reference group is Western Guangxi.

The regression results show:

1. Population size is a decisive factor: The permanent resident population coefficient is 0.17 (*p* < 0.01), indicating that for every 10,000 increase in permanent residents, the number of employees enrolled in medical insurance increases by an average of 0.17 million. This finding aligns with expectations—regions with larger populations have a larger employment base, resulting in correspondingly higher enrollment in employee medical insurance.
2. Aging rate fails significance test: The aging rate coefficient is −1.05 (*p* = 0.826), failing to reach statistical significance. This indicates that, after controlling for other factors, the degree of aging has no significant impact on employee medical insurance enrollment. This may be related to retired individuals retaining their medical insurance status within the enrolled population.
3. Regional dummy variables are insignificant: After controlling for permanent resident population, no significant differences in enrollment rates were observed between Northern, Southern, and Western Guangxi. This indicates that the regional enrollment disparities noted earlier (e.g., higher enrollment in Nanning and Liuzhou) are primarily driven by population size rather than geographic location itself.

This finding provides a causal explanation for regional disparities in Guangxi’s medical insurance funds: population size is the dominant factor determining the number of insured individuals. Regions with larger populations (such as Nanning) have more insured individuals and larger fund revenues; regions with smaller populations (such as Fangchenggang) have fewer insured individuals and limited fund revenues. As shown in Table 1, regions with higher aging rates (e.g., Guilin) have a larger proportion of retirees, resulting in greater fund expenditure pressures. It is precisely the combined effect of “fund revenue disparities stemming from population size differences” and “fund expenditure pressures arising from aging rate differences” that leads to the structural imbalance observed earlier: “funds lying idle in one area while depleted in another.”

### 3.2 Two models of provincial coordination of medical insurance

“Provincial coordination” refers to the institutional arrangement of integrating the basic medical insurance fund into a unified capital pool for centralized management with provincial administrative regions as the unit (12), which is intended to improve the fairness and sustainability of the system through fund mutual assistance, risk sharing and balanced allocation of resources.

At present, our country’s basic medical insurance provincial coordination mainly adopts two models, one is the “unified revenue and expenditure” model, and the other is the “adjustment fund” model. Both rely on the institutional framework of unified fund pools, and the difference lies in the significant difference in concentration and management methods. The unified revenue and expenditure model is a strategy of “complete unification and centralized management” (refer to Table 8). Now, most provinces started with the adjustment fund model and gradually transitioned to the unified revenue and expenditure model. In order to achieve scientific allocation of funds and balanced distribution of medical resources throughout the province, and ultimately promote a fairer and more sustainable medical insurance system.

**Table 8:** Comparison of two provincial pooling models: unified revenue-expenditure model vs. adjustment fund model.

Dimension	Adjustment fund model	Unified collection and expenditure model
Fund concentration	Partial concentration (limited to the adjustment of monetary funds)	All funds concentrated
Management authority	The region still retains the authority over fund management and expenditures	Provincial-level unified management and unified allocation
System positioning	Transitional model (Low Resistance to Reform)	Final mode (with greater fairness and risk resilience)
Whether to unify treatment	Existing of possible differences between regions	Achievement of unified funding and treatment standards in the province

In practice, provinces such as Sichuan and Shanxi are explicitly categorized in relevant literature as regions adopting the “ex-post adjustment fund” model (16). These provinces similarly employed this model as a transitional pathway toward achieving provincial-level pooling, implementing localized design and innovation in specific rules such as contribution ratios and deficit-sharing priorities.

#### 3.2.1 “Unified revenue and expenditure” model

“Unified revenue and expenditure” is the ultimate form of provincial coordination, and its core essence is to fully transfer key elements such as handling authority and fund revenue and expenditure to the provincial medical insurance administrative department. The provincial handling agencies undertake the important task of centralized management and unified accounting, and the medical insurance pooling funds of all districted cities in the province are remitted to the same fund pool, and the “six unifications” policy is implemented simultaneously, that is, unified policies and coverage, unified financing mechanisms, unified guarantee treatment, unified payment methods, unified fund management, and unified information systems (23). This model has significant advantages, allowing limited resources to be allocated and utilized more efficiently throughout the province. Greatly improve the efficiency of fund use and avoid idle and waste of funds; Enhance the fund’s resilience to risk (24), and from an economic efficiency perspective, it is expected to achieve fair and sustainable “Pareto improvements”^1^ (25).

However, the “unified income and expenditure” model also faces many challenges in the implementation process. It will affect the financial and administrative powers of provincial, municipal and county governments to make significant adjustments, and to a certain extent, it is easy to weaken the responsibilities of the municipal and county governments in medical insurance supervision and cost control (26). At the same time, it is also necessary to cross the triple hurdle of policy depression, reconstruction of rights and responsibilities and system upgrading, due to the differences in the payment base, reimbursement ratio, ceiling line and other aspects of each city, it is difficult to achieve unification in the short term; The rights and responsibilities of financing, payment, supervision, and coverage at the provincial, municipal and county levels need to be re-divided, and the coordination cost is high.

From a practical perspective, the reason why Beijing, Tianjin, Shanghai, Chongqing, Tibet, Qinghai, Ningxia, and other areas can directly implement the “unified revenue and expenditure” of medical insurance funds without going through the transitional stage of the “adjustment fund” is due to three factors., Regions with relatively small geographical areas and minor fiscal and policy disparities are more suited to the unified revenue and expenditure model. For example, Beijing, leveraging its flat “municipal-district” two-tier administrative structure, achieved complete unification of financing, benefits, and management quite early (for employee medical insurance in 2001), providing immediate and equitable protection for all insured individuals in the city. Second, the population size is small or evenly distributed, and the difference in funds is controllable. The total population of Tibet, Qinghai and Ningxia is limited and the mobility is low, and the differences in the degree of aging, disease spectrum distribution, and financing capacity between regions are within a controllable range.

Third, the reform started early, and the foundation of the “three unifications” of system, information and handling was solid. Tibet completed unified revenue and expenditure as early as 2000, and was the earliest provincial-level region in the country. Qinghai and Ningxia also completed the provincial unification of institutional policies, information systems, and handling procedures during the “13th Five-Year Plan” period, removing technical obstacles for the implementation of unified revenue and expenditure in one step (16, 27). With the comprehensive advantages of “flat administration, controllable population, and system first”, these regions have been able to skip the stage of “adjustment fund” and directly implement the “unified income and expenditure” of the medical insurance fund.

In stark contrast, Hainan’s eight-year practice is the epitome of the difficulty of promoting the “unified income and expenditure” model. Hainan started with the adjustment fund model in 2012, and promoted the provincial coordination of employee medical insurance and resident medical insurance in 2012 and 2015 respectively, narrowing the treatment gap year by year and unifying policy parameters, after years of policy running-in, system upgrading and rebalancing of rights and responsibilities, it was not until 2020 that the fund finally realized the unified revenue and expenditure management of the whole province (21). Since 2019, the province has implemented the “advance adjustment fund” model for employee medical insurance, and all localities have uniformly handed over the fund to the provincial level according to 30% of the actual collection amount of that year (increased to 50% in 2022), and the provincial level will then determine the adjustment amount of income from each coordinating area in advance according to the formula of “the number of insured people × the collection rate”, and plan to finally transition to unified revenue and expenditure (16). These practices show that the “unified revenue and expenditure” model is difficult to “do it in one step”. Despite this, unified revenue and expenditure are still the fundamental policy to eradicate the imbalance of regional funds, and it is also the long-term goal of provincial coordination of medical insurance.

#### 3.2.2 “Adjustment fund” model

The “adjustment fund” model is called “gradual coordination”, which builds a smooth transition channel for the promotion of provincial coordination of medical insurance. All regions continue to retain the existing fund pool and handling system, and only regularly release funds according to the established proportion at the provincial level to form a provincial “adjustment gold pool”. The provincial medical insurance bureau will then implement precise allocation to areas where the fund cannot meet the income and expenditure based on factors such as the degree of gap, payment ability, and performance results, so as to achieve “rich help the poor and fill the gap” (28). Under the adjustment fund model, the provincial medical insurance fund is still scattered in various places, and the provincial medical insurance department collects the adjustment fund every year according to a certain proportion of the overall fund of each city to fill the possible income and expenditure gap of the medical insurance fund, and effectively realize the diversification of medical insurance risks within the province (26). Because it does not touch the current financial, handling and supervision system, the reform resistance is small and the implementation is fast, which can alleviate the risk of fund bottoming out in underdeveloped areas in the short term, take into account efficiency and stability, and at the same time strive for a valuable time window for subsequent policy unification and information system integration. However, the adjustment fund is essentially an “out-of-pool adjustment,” which is a stopgap measure and cannot fundamentally eliminate the imbalance of regional funds.

In the process of medical insurance reform, Hainan Province has experienced a gradual promotion process from county (city) level to provincial level. In the 90s of the 20th century, there were 21 coordinating regions in Hainan Province, all of which were piloted with counties (cities) as social insurance coordinating units. However, due to the gap faced by the fund, poor mutual aid ability, high fund pressure, and with the arrival of the peak of aging, the limited fund payment capacity affects the enjoyment of the insured’s treatment, and at the same time, the disparity in medical treatment leads to the division of blocks, which cannot fully reflect the principle of “fairness and efficiency”, and it is urgent to implement provincial coordination of medical insurance (29).

In 2009, the “Recent Key Implementation Plan for Medical and Health System Reform (2009-2011)” proposed to “establish a risk adjustment fund system for basic medical insurance funds and improve the overall level of fund planning” (30). This policy points out the direction for the reform of medical insurance in Hainan Province. In the 21st century, with the increasing attention of the state to the improvement of the overall level of medical insurance, Hainan Province has ushered in new development opportunities in medical insurance reform. In 2012, Hainan Province decisively established a “provincial adjustment fund system” on the basis of the original municipal coordination, closely combining the central policy guidance and the actual situation of the local economy (12). This choice is not accidental, but the inevitable result of comprehensive consideration of many factors.

From a policy perspective, a number of national policies have pointed out the direction and reserved room for Hainan’s medical insurance reform. In particular, on February 14, 2012, the State Council officially issued the Plan for Deepening the Reform of the Medical and Health System During the 12th Five-Year Plan Period and Its Implementation Plan, which clearly stated that “efforts should be made to gradually establish a provincial-level risk adjustment fund system and actively promote provincial-level overall planning” (31). This plan mainly defines the phased goals, reform priorities and major tasks of the medical and health system reform from 2012 to 2015, and serves as a guiding document for deepening the reform of the medical and health system in the next 4 years. At the same time, as a special economic zone and an international tourist island, Hainan was entrusted by the central government with the special mission of taking the lead in innovating the medical insurance system and conducting pilot tests. In 2012, Hainan Province took the lead in implementing the provincial-level adjustment fund management model for the basic medical insurance fund for urban employees, providing policy support and opportunities for exploring a new medical insurance overall planning model.

From the analysis of the income and expenditure data of the medical insurance fund, it can be seen from the data in Table 3 that there are significant differences in the income and expenditure and balance level of the medical insurance fund between cities and counties in Hainan Province. Specifically, Haikou, Sanya, and Danzhou have high levels of income and expenditure and balance of medical insurance funds, showing strong sustainability of medical insurance funds. Wuzhishan City, Wenchang City, Qionghai City, Wanning City, these areas have a small scale of income and expenditure of medical insurance funds, but the balance level is relatively high, showing that their medical insurance funds are running relatively stable. The income and expenditure of the medical insurance fund in Dongfang City is moderate, but the balance level is low, showing a certain pressure on the operation of the medical insurance fund.

This imbalance provides the necessity for the implementation of the adjustment gold model. By adjusting medical insurance funds between different cities and counties, it can effectively solve the problems of high income and expenditure pressure and insufficient balance of medical insurance funds in some cities and counties, so as to ensure the sustainable operation of medical insurance funds and safeguard the medical security rights and interests of insured personnel. However, due to the disparity in balances in various regions, regions with large balances are often cautious about overall planning, fearing that their own interests will be damaged, thus forming certain resistance. For example, areas with large balances, such as Haikou City and Danzhou City, may have concerns about overall planning; In areas like Dongfang City, where the balance is low and it is easy to be unable to meet the expenditure, it is urgent to solve the sustainability problem of the medical insurance fund by improving the overall planning level.

In-depth analysis from the level of the number of insured people, as shown in Table 4, the participation rate varies significantly in different regions. Sanya has the highest insurance rate, reaching 102.57%, which is much higher than that of other regions, indicating that the enthusiasm for insurance in the region is extremely high, which may be related to the promotion of local policies or the strong awareness of residents’ participation in insurance; Haikou also has a higher insurance rate of 53.30%, indicating that the insurance situation in economically developed areas is better. In contrast, Wenchang City and Wuzhishan City have lower insurance rates of 17.85 and 31.71% respectively, indicating that the coverage of the medical insurance system in these areas is limited and the awareness of insurance needs to be improved. In addition, there are large differences in the proportion of active and retired people in different cities. For example, the proportion of active and retired people in Sanya is 67.73 (188,200/2,800), indicating that the proportion of retirees in the region is low and the pressure on the medical insurance fund is small; The proportion of active and retired people in Danzhou City is 2.65 (105,900/39,900), indicating that the proportion of retirees in the region is relatively high and the pressure on the medical insurance fund is greater.

This imbalance between the number of insured people and the proportion of on-the-job retirement between regions has led to insufficient income from medical insurance funds in some areas, making it difficult to cope with medical expenditures, while economically developed areas have surpluses of medical insurance funds. The overall reform of medical insurance implemented in 2012 adjusts the surplus of medical insurance funds in economically developed areas to economically underdeveloped areas through the adjustment fund model, alleviating the imbalance of medical insurance funds between regions, improving the overall level of protection, and promoting the fairness and sustainability of the medical insurance system. Therefore, the problems exposed by the population and medical insurance participation in 2011 provide an important decision-making basis for the overall reform of medical insurance in 2012, and promote the implementation of the reform to achieve the balanced development of the province’s medical insurance system.

From the institutional level, county-level finance has strong independence, slow progress of institutional reform, insufficient staffing, and lagging information system construction (32), all of which constitute practical obstacles to the implementation of the unified revenue and expenditure model. If the unified revenue and expenditure are directly implemented, it will not only face technical challenges, such as difficult information system integration and poor data sharing, but also encounter resistance at the interest level, such as the adjustment of local financial interests and the new distribution of medical institutions. These obstacles will greatly increase the complexity and difficulty of implementing reforms. In contrast, the adjustment gold model is more flexible and feasible. This model allows cities and counties to retain existing accounts and only need to hand over the balance funds at a rate of 10% (12), which will not put too much pressure on local finances, and can effectively realize the horizontal mutual assistance of funds. In this way, the provincial coordination of medical insurance funds can be gradually realized without touching the core interests of the local government, effectively smoothing out the friction that may arise in the reform process, and ensuring the smooth progress of the reform. Therefore, the adjustment fund model became an inevitable choice for Hainan Province at that time, laying a solid foundation for the further reform and improvement of the province’s medical insurance system.

Similarly, Jiangxi Province, serving as a representative case of the “ex-post adjustment fund” model, has established contribution rates for its employee and resident medical insurance provincial adjustment funds at 10 and 15%, respectively. The province has also implemented a fund allocation mechanism that incorporates an “incentive-based adjustment” component. For instance, 30% of the remaining provincial adjustment fund balance within a given period is allocated to reward pooling regions that did not apply for fund transfers. This approach effectively balances inter-regional fund disparities and stimulates local initiatives in fund collection and management. It provides significant practical reference for Guangxi in designing its own adjustment fund scheme, particularly for constructing incentive-compatible fund allocation rules.

### 3.3 Guangxi medical insurance provincial overall selection model

In 2021, the General Office of the State Council released the ‘14th Five-Year Plan for Universal Medical Security’, which clearly stated that provincial-level coordination should stick to the overall direction of “policy uniformity and standardization, fund balancing, improved hierarchical management, enhanced budget assessment, and better management services” (33). Among these, the mention of ‘fund balancing’ clearly indicates that the central government views the ‘transfer payment’ model as a starting point for provincial-level coordination. Compared to ‘centralized collection and expenditure’, the transfer payment system has advantages such as being easy to operate, controllable risks, and minimal disruption during reform, which many places have widely adopted as a ‘transition plan’ for provincial-level coordination. The usual path is to first spend 3 to 5 years running the ‘transfer payment’ to align responsibilities and policies between provinces and cities, before smoothly transitioning to the full ‘centralized collection and expenditure’ phase. The central government’s top-level design combined with local pilot programs proves that the transfer payment model is a practical choice that balances inter-regional solidarity and risk management.

Guangxi is facing multi-dimensional and complex challenges in promoting the provincial coordination of medical insurance. From the perspective of policy differences, there are significant regional differences in medical insurance policies, treatment standards and fund management in Guangxi, which are closely related to the local economic development level, demographic characteristics and distribution of medical resources. If the unified revenue and expenditure model is forcibly implemented, it is easy to cause the problem of poor policy connection, which in turn increases the cost of policy implementation and weakens the effect of policy implementation. In contrast, the adjustment fund model shows greater flexibility, which can gradually promote overall planning on the basis of maintaining the relative stability of local medical insurance policies, effectively reduce the impact of policy adjustments, and is conducive to the smooth transition of medical insurance policies.

From the perspective of medical insurance fund revenue and expenditure, the income and expenditure of Guangxi employee medical insurance fund (including maternity insurance) in 2023 will show a significant imbalance. According to the “2023 Guangxi Medical Security Development Statistical Bulletin” released on the official website of the Guangxi Medical Insurance Bureau, the income of the medical insurance fund reached 38.870 billion yuan that year, an increase of 6.23% over the previous year. Among them, the income of the overall fund was 26.866 billion yuan, a significant increase of 20.32% year-on-year; In terms of expenditure, the total fund expenditure was 34.522 billion yuan, an increase of 19.02% year-on-year. Specifically, the overall fund expenditure was 20.216 billion yuan, an increase of 19.37%; On the other hand, the current balance of the overall fund was 6.650 billion yuan, and the cumulative balance reached 37.736 billion yuan. Behind this difference in income and expenditure is the imbalance in the level of economic development between regions, industries and enterprises.

As shown in Table 2, in relatively economically developed areas such as Nanning, the income of the employee medical insurance fund is 7.873 billion yuan, the expenditure is 7.272 billion yuan, and the cumulative balance is 13.156 billion yuan. For example, the income of the employee medical insurance fund in Guilin City is 3.610 billion yuan, the expenditure is 4.079 billion yuan, and the cumulative balance is only 28 million yuan. The lack of cumulative balance data of employee medical insurance funds in some areas such as Wuzhou City and Beihai City may also reflect certain situations in the management of their medical insurance funds or data statistics. This difference in the income, expenditure and balance of employee medical insurance funds in different regions reflects the differences in the level of economic development, the distribution of medical resources, and the medical needs of insured personnel between regions. Although the income of medical insurance funds in economically developed areas may be relatively sufficient, due to the concentration of medical resources and the strong medical demand of insured personnel, the expenditure pressure is huge; In economically underdeveloped areas, the income of medical insurance funds is already limited, and medical resources are relatively scarce, making it difficult to fully meet the medical needs of insured personnel.

At the same time, from the comparison of employee and resident medical insurance funds, the income and expenditure of residents’ medical insurance funds in each city are also different. For example, the income of the Guigang Residents’ Medical Insurance Fund was 4.843 billion yuan, the expenditure was 4.257 billion yuan, and the cumulative balance was 6.158 billion yuan; the income of the Yulin Residents’ Medical Insurance Fund was 5.767 billion yuan and the expenditure was 5.644 billion yuan. The difference in the economic status of enterprises in different industries will be reflected in the income and expenditure of the employee medical insurance fund, while the income and expenditure of the resident medical insurance fund are more affected by factors such as regional population structure, residents’ income level, and medical security policies, which also makes the stability of the income and expenditure of the medical insurance fund affected by many aspects. In this context, the implementation of the adjustment fund model is particularly necessary. It can effectively balance the difference in income and expenditure of medical insurance funds between regions and different types of medical insurance, alleviate the pressure on the income and expenditure of medical insurance funds through reasonable adjustment of funds between different entities, enhance the anti-risk ability of the fund, protect the legitimate rights and interests of insured personnel, and promote the sustainable development of medical insurance funds and the realization of social equity.

From the perspective of the insured population structure, there are significant differences in the structure and demographic structure of the insured population in different regions of Guangxi, which have a profound impact on the distribution and use efficiency of medical insurance funds. As shown in Table 4, from the perspective of the structure of the insured population, the number of employees and residents in each city is significantly different. For example, the number of employees insured by medical insurance in Nanning is 1,658,000, the number of resident medical insurance participants is 5,785,900, while the number of employees insured by medical insurance in Fangchenggang City is 177,900, and the number of resident medical insurance participants is 770,900. This difference leads to different demand and spending pressures for medical insurance funds. In areas with a large number of employee medical insurance participants, the demand for medical insurance funds is large and the expenditure pressure is also great; in areas with a small number of insured people, the demand for funds is relatively small, and funds may be idle. In addition, the degree of aging in different regions is different, with the proportion of the older population aged 60 and above in Guilin being 21.20%, which is higher than the average in Guangxi (17.45%), while Fangchenggang City is only 14.70%. In areas with a high degree of aging, the proportion of retired employees is high, and the expenditure pressure of medical insurance funds is high; in areas with a low degree of aging, the pressure on medical insurance fund expenditure is small and the funds are relatively abundant. This difference makes it difficult for the existing medical insurance fund allocation methods to accurately match the needs of different regions, resulting in tight medical insurance funds in some areas and idle funds in some areas.

In this context, the existing medical insurance fund allocation methods are difficult to accurately match the demographic differences in different regions, which can easily lead to the coexistence of the medical insurance fund gap in aging cities and the idle funds in young areas. Furthermore, Guangxi’s medical resources show the characteristics of “urban centralization,” and the average hospitalization cost of tertiary hospitals is as high as 12,835 yuan, which is 4.6 times that of medical institutions at level 1 and below (2,796 yuan) (18). This distribution method can neither guide medical resources to sink to the grassroots level through fund allocation, nor can it effectively alleviate the contradiction between “too high proportion of high-cost diagnosis and treatment” and “insufficient utilization rate of grassroots services”. Therefore, the adjustment fund model is more flexible and targeted, and it can dynamically adjust the allocation of funds between regions according to key indicators such as the proportion of the older population and the ratio of on-the-job retirement. For example, the adjustment quota will be increased for cities such as Liuzhou and Guilin, which have a serious aging population, to ensure their ability to pay for medical insurance. At the same time, through policies and measures such as financial support for the construction of primary medical institutions and increasing the reimbursement ratio of grassroots medical treatment, we will promote the rational diversion of patients and reduce the phenomenon of excessive diagnosis and treatment in tertiary hospitals. In this way, it can not only optimize the resource allocation of aging areas in cities such as Liuzhou and Guilin, but also improve the efficiency of the use of medical insurance funds in young areas such as Baise and Hechi, realize the linkage balance of “population-resources-fund,” and better meet the actual needs of Guangxi’s current population structure differentiation and imbalance of medical resources.

Combined with the structure of the insured population and the population structure, the comprehensive analysis shows that there are significant differences in the allocation and use efficiency of medical insurance funds in different regions of Guangxi. For example, Nanning, as a large population city, has a large number of employees and residents’ medical insurance, the aging rate is at a moderate level, and the demand for medical insurance funds is large, but the efficiency of fund use can be improved through scale effects. While Guilin and Liuzhou have a high degree of aging, the number of employees participating in medical insurance is relatively small, and the pressure on medical insurance fund expenditure is high, so more financial support is needed. Fangchenggang City has a low aging degree and a relatively abundant medical insurance fund, but it needs to improve the efficiency of the use of medical insurance funds through policy guidance. This difference needs to be addressed through a more flexible adjustment mechanism to ensure the rational allocation and efficient use of medical insurance funds.

From the perspective of handling services, the differences in handling services also restrict the promotion of provincial coordination of medical insurance. There are obvious differences in the process, standards, information systems and other aspects of medical insurance handling services in various places, and the unified revenue and expenditure model requires the whole province to unify standards and processes, which is difficult to achieve in the short term, and requires large-scale training and system upgrades, which not only consumes time and costs, but also may lead to a lack of motivation to control costs and improve the efficiency of fund use in some areas. In contrast, the adjustment fund model can incentivize all localities to improve the efficiency of fund use and control the unreasonable growth of expenses by setting adjustment conditions and ratios. In recent years, the Guangxi medical insurance department has made every effort to improve and optimize the five-level medical insurance management service system of autonomous regions, cities, counties, townships (streets) and villages (communities) around "listization, standardization, informatization and specialization". According to the "National List of Government Services Handled by Medical Security (2023 Edition)", the Medical Insurance Bureau of the Autonomous Region has reorganized and formed a list of 43 medical insurance handling services in 13 categories based on the actual situation in Guangxi, and promoted the non-discriminatory handling of government service matters handled by medical insurance in the region, making it simpler and clearer for the masses to handle affairs. In addition, Guangxi has also actively promoted “Internet” medical insurance services, continuously enriched application scenarios, and medical insurance codes are becoming more and more widely used in hospitalization, outpatient and other links of designated medical institutions, realizing convenient operations such as registration, drug collection, reporting, inspection and testing, and medical insurance payment, and the insured people in the region have obtained more high-quality, convenient and efficient medical insurance services (34).

In terms of fund income and expenditure risk control, the growth rate of expenditure of Guangxi employee medical insurance fund (including maternity insurance) in 2023 (19.02%) is significantly higher than the growth rate of income (6.23%) (18), and the pressure on fund revenue and expenditure continues to increase. From the data in Table 3, this pressure varies significantly between different regions. For example, the income of the Nanning Employee Medical Insurance Fund was 7.873 billion yuan, the expenditure was 7.272 billion yuan, and the cumulative balance reached 13.156 billion yuan, and the fund was relatively healthy; while the income of the Guilin Employee Medical Insurance Fund was 3.610 billion yuan, the expenditure was 4.079 billion yuan, and the cumulative balance was only 28 million yuan, facing greater pressure. The expenditure of the employee medical insurance fund in Wuzhou, Yulin, Baise and Hechi is close to or exceeds the income, indicating that the pressure on revenue and expenditure is greater, and the cumulative balance data in some areas is missing. This difference shows that some regions have high fund balances, while others face a tight fund situation. Regions with high balances may be reluctant to participate in unified revenue and expenditure, fearing that their own interests will be damaged.

In this context, the adjustment fund model can diversify risks, enhance the anti-risk ability of the medical insurance fund, and provide a strong guarantee for the sustainability of the medical insurance fund. Through the adjustment fund model, the fund allocation between regions can be dynamically adjusted according to the income and expenditure of each region and the characteristics of the population structure. For example, for areas with high fund balances, the amount of adjustment funds can be appropriately reduced; for areas with greater pressure on fund expenditure, the allocation amount of adjustment funds can be increased to alleviate their income and expenditure pressure. This model can not only effectively meet the needs of regions with high aging and high medical expenses, but also encourage regions to reasonably control the growth of medical expenses and improve the efficiency of the use of medical insurance funds. At the same time, the adjustment fund model can also promote the rational diversion of patients, reduce the phenomenon of excessive diagnosis and treatment in tertiary hospitals, and further optimize the allocation of medical resources through policy measures such as supporting the construction of primary medical institutions and increasing the proportion of reimbursement for grassroots medical treatment.

In view of the above-mentioned multiple structural problems faced by the provincial coordination of medical insurance in Guangxi, it is recommended to adopt the strategy of “active and prudent and step-by-step progress”. Establish a “adjustment fund” model in advance, and set up an autonomous region-level adjustment gold pool as the core carrier. Through the scientific assessment and analysis of the income and expenditure of medical insurance funds in various places, determine the reasonable adjustment standards and ratios, and implement “precise compensation” for gap areas to ensure that these areas pay medical insurance benefits on time and in full, and protect the basic rights and interests of insured personnel; The integration of the information system is the key to the efficient operation of the adjustment fund model, establish a unified medical insurance information system in the province, realize real-time data sharing and interconnection, and provide accurate data support for the collection, distribution and management of the adjustment fund. At the same time, we will speed up the connection between payment and treatment policies to ensure that insured personnel in various regions are fair and consistent in terms of payment standards and treatment enjoyment, and reduce contradictions and problems caused by policy differences.

From the perspective of urban–rural integration, the structural imbalance in Guangxi’s medical insurance funds also manifests in disparities in resource allocation between urban and rural areas. As shown in Table 4, the number of residents enrolled in urban–rural resident medical insurance far exceeds that of employees enrolled in urban employee medical insurance across all cities (e.g., Nanning has 5.7859 million residents enrolled in resident medical insurance, which is 3.5 times the 1.658 million enrolled in employee medical insurance). However, the sustainability of the resident medical insurance fund faces greater challenges. In 2023, the cumulative surplus of Guangxi’s resident medical insurance fund covered 11.51 months of expenditures. While this figure slightly exceeds the reasonable upper limit, some coordinated regions have already experienced current expenditures exceeding current revenues, forcing them to draw upon accumulated surpluses.

The uneven distribution of medical resources between urban and rural areas further exacerbates this contradiction. Tertiary hospitals are primarily concentrated in central cities like Nanning and Liuzhou, where the average hospitalization cost reaches 12,835 yuan—4.6 times that of general hospitals. The “siphoning effect” generated by rural residents seeking medical care in cities not only diverts funds to urban areas but also increases the indirect medical burden on rural residents. Tian et al. (8) demonstrated that the direct settlement policy for cross-provincial medical treatment has a significant positive impact on the health of urban–rural migrant populations, though this effect relies on higher-level coordination arrangements. Yan (7) found that medical insurance integration policies reduced benefits for urban residents while increasing benefits for rural residents, with effects varying according to regional economic development levels.

Therefore, in advancing provincial-level coordination, it is essential to strengthen primary healthcare service capabilities at the grassroots level to prevent further concentration of medical resources in urban areas. Only by supporting rural regions and primary healthcare institutions through provincial-level redistribution mechanisms can we truly achieve healthcare equity from an integrated urban–rural perspective.

### 3.4 Suggestions for promoting provincial coordination of basic medical insurance in Guangxi

Based on the above analysis, Guangxi must fully consider the unique characteristics of multi-ethnic regions and the requirements for integrated urban–rural development when advancing provincial-level coordination. Specifically, the design of the provincial coordination plan should embody the following two principles: First, provide appropriate fund to concentrated areas of ethnic autonomous counties, enhancing their risk-resilience through provincial-level adjustment mechanisms. Second, promote equitable access to medical services between urban and rural areas by optimizing resource allocation, ensuring that rural residents do not experience a reduction in actual benefits under provincial coordination.

#### 3.4.1 Establishing a “dual-track adjustment funds”

The basic medical insurance system in Guangxi currently encompasses both employee insurance and resident insurance, which exhibit significant differences in their financing mechanisms and benefit levels, having, respectively, formed independent fund pools. Therefore, provincial-level coordination should establish two distinct channels for fund adjustment: one for employee insurance and one for resident insurance. Specifically, the adjustment funds for employee and resident insurance should be deposited separately into the financial dedicated accounts of the Autonomous Region’s medical insurance fund, where they will be independently accounted for and operated in a closed manner. This arrangement ensures that the funds are used specifically for their intended purposes, isolates risks, safeguards fund security, and enhances the efficiency of the adjustment funds.

#### 3.4.2 Establish a differentiated adjustment mechanism for multiethnic regions and urban–rural integration

Given Guangxi’s multi-ethnic composition and significant urban–rural disparities, it is recommended to incorporate differentiated adjustment factors into the design of the transfer payment model:

1. Ethnic Autonomous County Adjustment Coefficient: When calculating the proportion of municipal contributions to provincial adjustment funds or the allocation of provincial adjustment funds, provide appropriate to cities with a high concentration of ethnic autonomous counties (Liuzhou, Laibin, Hechi, Baise, Guilin). Specifically, a weighted approach can be adopted, incorporating factors such as the number of ethnic autonomous counties and the proportion of ethnic minority populations into the calculation formula, ensuring that fund adjustments more precisely benefit ethnic regions.
2. Rural Resident Benefit Protection Mechanism: Provincial redistribution funds should prioritize ensuring stable medical insurance payments for rural residents, preventing the diversion of rural healthcare resources due to fund deficits in individual cities. Concurrently, special funds may be established to support primary healthcare facility development, enhancing medical service accessibility in rural areas and mitigating the “siphoning effect” caused by rural residents seeking urban medical care at its source.
3. Dynamic Monitoring and Evaluation: Establish a fund operation monitoring indicator system from the perspectives of multi-ethnic regions and urban–rural integration. Regularly assess the actual impact of provincial pooling on different regions and groups, promptly adjust redistribution policies, and ensure the achievement of “Pareto improvement”—where municipal and county interests remain unaffected, provincial mutual aid capacity is enhanced, and actual benefits for rural residents are not diminished.

#### 3.4.3 Extraction and use of “adjustment funds”

##### 3.4.3.1 Principles of extracting adjustment funds

The withdrawal of adjustment funds should take into account the stability of the fund pool size and the positive incentives for the coordinating regions, and the withdrawal ratio should be flexibly adjusted according to the operation of local medical insurance funds and policy objectives. For example: “Jiangxi, Shanxi, Ningxia and other provinces adopt the ex post adjustment fund model, and the proportion of provincial adjustment ranges from 3% to 15%, of which Jiangxi has the highest adjustment ratio, and the proportion of employee medical insurance and resident medical insurance is 10% and 15% respectively (16). For example, after implementing this model, Jiangxi Province effectively managed fund surpluses and deficits at the provincial level without triggering payment risks in any locality, while maintaining local collection and management enthusiasm, thereby accumulating administrative experience for transitioning to a higher pooling level. It not only ensures the replenishment of provincial “reservoirs” but also avoids excessive withdrawal of local funds. It is recommended that on the basis of scientific calculation, the Guangxi Medical Insurance Bureau should take “benchmark ratio + flexible adjustment” as the core, set 3–15% as the overall range, dynamically include key indicators such as the number of months of fund payment, balance rate, support ratio and expenditure growth rate every year, implement rolling calculation and file extraction, and form a precise incentive mechanism of “gap sharing and savings with rewards.”

##### 3.4.3.2 Rules for the use of adjustment funds

The use of adjustment funds should not only resolve the structural imbalance of the medical insurance fund, but also establish an incentive mechanism for medical security reform (35). Taking Jiangxi Province as an example, three distribution methods are established in the early stage of system construction: one is normal adjustment, for the cumulative balance of the employee medical insurance fund for less than 9 months and the cumulative balance of the resident medical insurance fund for less than 6 months, the provincial adjustment fund will be supplemented by the normal adjustment method; the second is emergency adjustment, which is used to deal with the fund operation risks caused by public health emergencies and major policy adjustments, and the provincial adjustment fund will be adjusted according to the actual application of each coordinating district; The third is to incentivize the adjustment, and the medical insurance funds recovered by the supervision of medical treatment in different places in the province will be rewarded and returned according to the ratio of 40% of the place of medical treatment and 40% of the insured place, and at the same time, for the overall planning area that has not obtained normal adjustment and emergency adjustment, combined with the effectiveness of its medical insurance work, the contribution of the provincial adjustment fund and other factors, the remaining provincial adjustment fund will be rewarded and returned according to the proportion of 30% of the remaining provincial adjustment fund in the current period (36). Drawing on the above experience, Guangxi can clearly divide the provincial transfer fund into three use channels, so as to achieve the trinity of “filling the gap, rewarding the advanced, and preventing risks” to ensure the rational allocation and use of medical insurance funds.

###### 3.4.3.2.1 Deficit adjustment (regular adjustment)

For the co-ordinated areas where the cumulative balance of the co-ordinating fund at the end of the year can be paid for less than 6 months after the payment of the adjustment fund this year, the provincial adjustment fund will be supplemented to 6 months according to the type of insurance. If there is a gap in the overall fund of each coordinating region in the current year, the accumulated balance of the co-ordinating region over the years will be used to pay first; when the balance over the years is insufficient to pay, the adjustment fund will be used again. The autonomous region will comprehensively determine the gap sharing ratio based on factors such as the on-the-job retirement ratio, the fund collection rate, the completion of the insurance expansion task, the final accounts, and the performance evaluation of the use of the fund, which will be jointly borne by the autonomous region-level adjustment fund and the coordinating regional government.

###### 3.4.3.2.2 Assessment adjustment (incentive adjustment)

After the deficit adjustment, all remaining funds will be transferred to the incentive pool. The Medical Insurance Bureau of the Autonomous Region, together with the Department of Finance, takes quantitative indicators such as insurance expansion, fund collection, expenditure control, and regulatory effectiveness as the core, and implements “more work, more gains, and excellent performance” for non-deficit areas, so that regions that can save money can share the dividends of reform, form a clear orientation of rewarding diligence and punishing laziness, and encourage all regions to actively participate in medical insurance reform.

###### 3.4.3.2.3 Urgent adjustment

In the event of sudden public health incidents or significant policy adjustments leading to insufficient fund payment capabilities in coordinated areas, provincial adjustment funds may be urgently allocated according to application circumstances. An application for urgent funding may be applied for on a ‘case-by-case’ basis. The conditions for adjustment, approval processes, and repayment methods will be separately formulated by the Autonomous Region’s Medical Insurance Bureau in conjunction with the Department of Finance, ensuring that assistance is provided in emergencies but not for alleviating poverty, and that the use of funds is regulated.

#### 3.4.4 Guidance of medical insurance to transition towards “health risk” from provincial-level coordination

Whether Guangxi ultimately adopts a “unified collection and expenditure” model or a “adjustment fund” model, the institutional concept of provincial-level planning should shift from the traditional “risk management-oriented” approach to a “health risk-oriented” approach. This means that the extraction, distribution, and utilization of the adjustment fund should no longer be limited to the traditional financial perspective of “controlling expenditures and reducing costs,” but should instead allow the fund to truly “follow health needs.” Specifically, a dynamic weight model should be constructed centered on multi-dimensional risk factors such as aging population rates, chronic disease prevalence, changes in disease spectrum, gradient allocation of medical resources, and differences in economic development. This will enable precise redistribution of the medical insurance fund. Consequently, the flow of funds will resonate in sync with health needs, narrowing the disparity in protection between regions, while pressing resources towards grassroots and health management, ultimately enhancing the fairness and efficiency of the fund, and ensuring that the health rights and interests of insured individuals are effectively realized.

#### 3.4.5 Taking the construction of information digitalization as the provincial overall plan to erect pillars and beams

The construction of information digitalization is an important support for the provincial overall planning of medical insurance, which should be promoted from the following three aspects: first, the information management of medical insurance payers and the establishment of a comprehensive information database of actual medical insurance payers, covering the age, gender, economic income and other basic information of the insured in each coordinating area. This information is the basis for the implementation of the adjustment fund system, which can provide data support for the accurate allocation of the medical insurance fund and ensure the fairness and rationality of the use of the fund. The second is health risk information management, which builds a health risk information platform for insured persons to record health-related data such as historical medication, hospitalization information, and disease history of insured persons. This information is the core basis for the medical insurance fund to “follow the health risks”, which can help the medical insurance department better understand the health needs of various places, so as to realize the scientific allocation and accurate use of the medical insurance fund. The third is information sharing and coordination between regions, strengthening the informatization construction between coordinating regions and autonomous regions, and realizing the information interconnection between medical service providers and medical insurance agencies. By establishing an information sharing mechanism between local governments and between local governments and autonomous regions, we can break down information islands, improve the efficiency and service quality of medical insurance management, and provide a strong guarantee for the smooth implementation of provincial coordination of medical insurance.4. This mirrors the logic behind Zhejiang Province’s ‘Golden Insurance Project,’ which established a unified provincial information framework that was crucial for its successful provincial-level pooling.

## 4 Discussion

### 4.1 Key findings and policy implications

This study reveals significant structural imbalances in Guangxi’s medical insurance funds, with coexisting excessive balances and deficit risks across different regions. The aforementioned siphoning effect in cross-regional medical treatments further exacerbates regional disparities, highlighting the urgency of promoting provincial-level overall planning. These findings align with existing research on medical insurance fund management challenges in China (37, 38), while providing specific evidence for Guangxi’s context. The adjustment fund model emerges as a pragmatic transitional approach, balancing reform needs with implementation feasibility.

### 4.2 Theoretical contributions and practical significance

The research contributes to the theoretical understanding of medical insurance fund management by comparing the applicability of different provincial-level overall planning models in regional disparity contexts. The findings support the principal-agent theory perspective in designing incentive-compatible mechanisms during system transition (39, 40). Practically, the study provides concrete implementation suggestions for Guangxi, including establishing separate adjustment fund pools for employee and resident insurance, formulating scientific fund extraction and usage rules, and developing transition paths toward unified revenue and expenditure management.

### 4.3 Comparative analysis with other regions’ experiences

The experiences of Hainan and Jiangxi provinces in implementing provincial-level overall planning offer valuable lessons for Guangxi (41, 42). Hainan’s success with the unified revenue and expenditure model demonstrates the importance of strong provincial-level coordination capabilities, while Jiangxi’s gradual approach highlights the value of phased implementation. However, Guangxi’s particular regional disparities and economic development level necessitate tailored solutions rather than direct replication of other regions’ models.

Furthermore, situating this study’s analysis of Guangxi within the broader context of western multi-ethnic provinces in China helps deepen the understanding of the applicability of the ‘adjustment fund’ model. Although the 16 provinces covered in the referenced literature (16) do not include Yunnan and Guizhou, its analytical framework offers important insights. The literature points out that provinces with “more complex interest structures” or “significant inter-regional policy disparities” (such as Sichuan and Shandong) tend to adopt the ‘ex-post adjustment fund’ model as a transitional pathway to achieve gradual policy unification and risk balancing (16). Yunnan, Guizhou, and Guangxi all belong to western multi-ethnic regions, sharing common structural constraints such as intra-provincial development imbalances, concentrated ethnic minority populations, and uneven distribution of medical resources. Based on this, it is reasonable to infer that these two provinces, when advancing provincial-level pooling, may similarly face complex interest structures akin to the “provinces with significant policy disparities” described in literature (16). Consequently, they would prudently assess the immediate feasibility of “unified revenue and expenditure” and tend to adopt or explore some form of fund adjustment mechanism to mitigate regional risks in the early stages of reform while motivating local initiatives. This logic indirectly validates that, under similar structural constraints, the ‘adjustment fund’, as a gradual reform path, possesses inherent rationale that extends beyond individual provincial cases, offering broader typological reference value. Future research could draw on the policy tool analysis method from literature (16) to conduct systematic comparative case studies on western multi-ethnic provinces like Yunnan, Guizhou, and Guangxi, thereby providing more precise policy references.

### 4.4 Research limitations and future directions

This study has several limitations that suggest directions for future research. First, data unavailability. Due to disclosure limitations in municipal statistical bulletins, data on the accumulated balance of urban employee basic medical insurance funds for Wuzhou, Beihai, Yulin, and Baise were unavailable. This may lead to some bias in estimating the total scale of medical insurance funds in Guangxi and may affect the accuracy of regional disparity measurements. These four missing samples were excluded from the relevant analyses, but the reduced sample size may impact the representativeness of the results. Future research could obtain more complete data through official channels or employ multi-source data (e.g., medical institution reports, tax collection data) for cross-validation to achieve more precise analytical results.

Second, cross-sectional data limitations. This study primarily analyzes cross-sectional data from 2023, which cannot capture the dynamic evolution of regional disparities in medical insurance funds over time. Subsequent studies could collect multi-year panel data and employ more sophisticated econometric models to analyze the evolutionary patterns of regional disparities and their influencing factors, providing stronger evidence for the dynamic adjustment of provincial-level pooling policies.

Third, case selection limitations. This study uses Hainan as a typical case for policy, but differences between Hainan and Guangxi in terms of administrative area, population size, and economic structure require further validation of the applicability of Hainan’s experience. Future research could select more comparable provinces (such as Jiangxi and Shanxi, which also adopted the adjustment fund model) for multi-case comparisons to enhance the generalizability of the findings.

Fourth, reliance on secondary data. The research primarily uses secondary data, which may not capture all implementation details and stakeholder perspectives. Qualitative approaches such as interviews with policymakers and medical institution representatives could provide deeper insights.

Fifth, geographic focus. The focus on Guangxi may limit the generalizability of findings, suggesting the need for comparative studies across different provincial contexts.

Thus, Guangxi’s proposed pathway—“adjustment fund transition → gradual policy and IT system unification → evolution towards unified revenue and expenditure”—is well-precedented by multiple provinces. The key is to execute a refined design within this framework, incorporating features for multi-ethnic and urban–rural integration.

## 5 Conclusion

This study demonstrates the necessity and urgency of promoting provincial-level overall planning of basic medical insurance in Guangxi. The significant structural imbalances in medical insurance funds, coupled with the challenges of cross-regional medical treatments, necessitate immediate policy intervention. The implications of this study extend beyond Guangxi. Its core value lies in providing empirical evidence and a model selection rationale for regions sharing the common constraints of ‘multi-ethnic concentration, significant urban–rural gaps, and regional development imbalances,’ exploring a feasible, gradual reform path with lower resistance. The adjustment fund model is recommended as a pragmatic transitional approach for Guangxi, given its flexibility and lower implementation resistance compared to the unified revenue and expenditure model. The proposed implementation framework includes establishing dual-track adjustment funds, developing scientific fund management rules, promoting digital infrastructure construction, and designing a clear transition path toward unified revenue and expenditure management. These measures will optimize fund allocation efficiency, enhance system sustainability, and ultimately improve medical security equity for all residents in Guangxi.

## Data Availability

The original contributions presented in the study are included in the article/supplementary material, further inquiries can be directed to the corresponding author/s.

## Footnotes

1 “Pareto improvement” is an idealized criterion of economic efficiency. As long as none of the welfare of one party has declined as a result of the reform, and at least one party has become better, the change is considered a Pareto improvement. Taking the provincial overall planning of medical insurance as an example, after the unified revenue and expenditure, the funds originally precipitated in City A were used by the whole province, and the treatment of insured people in City A did not decrease but increased; The original gap in City B has been filled in time, and the benefits of the insured have been significantly improved; And no one is damaged in the whole process, which constitutes a Pareto improvement. If the reform brings overall benefits and damages some people, it is necessary to achieve “Kardo improvement” through the compensation mechanism. Although Pareto improvement is difficult to fully achieve in practice, it still provides an optimal goal worth pursuing for policy design.

## References

[1] Mathauer I Saksena P Kutzin J. Pooling arrangements in health financing systems: a proposed classification. Int J Equity Health. (2019) 18:198. doi: 10.1186/s12939-019-1088-x, 31864355 PMC6925450

[2] Flourence M Jarawan E Boiangiu M el Yamani FEK. Moving toward universal health coverage with a national health insurance program: a scoping review and narrative synthesis of experiences in eleven low- and lower-middle income countries. PLOS Global Public Health. (2025) 5:e0003651. doi: 10.1371/journal.pgph.0003651, 39787117 PMC11717203

[3] Chuangzhou X. A Study on the Control of Medical Expenses in the New Rural Cooperative Medical Scheme [D]. Yangling, China: Northwest A&F University (2011).

[4] Dong B. The promotion of pooling level of basic medical insurance and participants' health: impact effects and mediating mechanisms. Int J Equity Health. (2023) 22:105. doi: 10.1186/s12939-023-01927-1, 37287060 PMC10246363

[5] Hu F Zhang X Wang L Li Y Chen S Liu Z . Enhancing pooling levels strengthens the risk resilience of healthcare insurance: empirical evidence from Gansu, China. BMC Public Health. (2024) 24:1129. doi: 10.1186/s12889-024-18623-2, 38654172 PMC11040927

[6] Salari P Crivelli L. The inequity of the Swiss health care system financing from a federal state perspective. Int J Equity Health. (2014) 13:17. doi: 10.1186/1475-9276-13-17, 24524216 PMC3926944

[7] Yan J. Medical insurance integration and spatial effects in China: evidence from quasi-natural experiments and linked administrative data. BMC Health Serv Res. (2025) 25:805. doi: 10.1186/s12913-025-12946-9, 40474186 PMC12139253

[8] Tian J Chen Z Wang Y Zhu Y. Does the trans-provincial immediate reimbursement reduce health gap between urban and rural floating population? Evidence from China. BMC Public Health. (2025) 25:1826. doi: 10.1186/s12889-025-23027-1, 40382571 PMC12084940

[9] Qinlei Z. A Study on the Division of Responsibilities and Expenditure Obligations in Basic Medical Insurance. Shanghai, China: Shanghai University of Finance and Economics (2021).

[10] Zhang A Nikoloski Z Mossialos E. Does health insurance reduce out-of-pocket expenditure? Heterogeneity among China's middle-aged and elderly. Soc Sci Med. (2017) 190:11–9. doi: 10.1016/j.socscimed.2017.08.005, 28823943

[11] Huang L Yang D Yao L Liu Z Wu W. Guangxi's rural health insurance scheme: evidence from an ethnic minority region in China. Rural Remote Health. (2013) 13:2454. doi: 10.22605/RRH2454, 23574284

[12] Yuan T. Scientific connotation and path optimization of provincial overall planning of basic medical insurance. Acad J Zhongzhou. (2023) 12:85–94. doi: 10.3969/j.issn.1003-0751.2023.12.012

[13] The State Council of the People's Republic of China. Xi Jinping: Hold High the Great Banner of Socialism with Chinese Characteristics and Strive for the Comprehensive Construction of a Modern Socialist Country—Report to the 20th National Congress of the Communist Party of China [EB/OL]. Available online at: https://www.gov.cn/xinwen/2022-10/25/content_5721685.htm (2022)

[14] China National Radio. Relevant to People's Livelihood Security, the Latest Deployment of the Third Plenary Session of the 20th Central Committee [EB/OL]. (2024). Available online at: https://news.cnr.cn/native/gd/20240723/t20240723_526809701.shtml (Accessed September 5, 2025).

[15] China Pharmaceutical Innovation Promotion Association. Draft Law on Medical Security Released, Five Aspects Worth Paying Attention To [EB/OL]. (2025). Available online at: https://www.phirda.com/artilce_39522.html?module=trackingCodeGenerator (Accessed January 12, 2026).

[16] Zhu XL Wang YH. Comparative analysis and optimization of provincial-level pooling policies for basic medical insurance in selected provinces of China. China Health Insur. (2024) 10:29–34. doi: 10.19546/j.cnki.12-1143/d.2024.10.006

[17] Wang ZW Yao J. The medical insurance dilemma under the principal-agent theory: a case study of the basic medical insurance for urban employees. Insur Stud. (2023) 11:104–18. doi: 10.3969/j.issn.1004-3306.2023.11.009

[18] Guangxi Medical Security Bureau. Statistical Communique on the Development of Medical Security in Guangxi [EB/OL]. (2023). Available online at: https://ybj.gxzf.gov.cn/zfxxgkzl/fdzdgknr/tjxx/t18936048.shtml (Accessed October 18, 2025).

[19] Nanchang Municipal People's Government. Guiding Opinions on Further Strengthening the Management of Basic Medical Security Funds [EB/OL]. (2009). Available online at: https://www.nc.gov.cn/ncszf/zcwjb/200907/3ef6726c2ffc4f86a6528b115dae0383.shtml (Accessed November 3, 2025).

[20] Xu ZY Xu W. Does the "no-filing" policy for cross-province medical services affect the choices, medical expenses, and insurance funds for urban employees' basic medical insurance?—an empirical study based on a difference-in-differences model. Chin J Health Policy. (2021) 14:36–41. doi: 10.3969/j.issn.1674-2982.2021.14.005

[21] Zhou JH Xing LY Wang Y Chen Z Li M Wu Q . The influence of provincial-level medical insurance pooling and medical service quality on patient healthcare behavior in county-level medical institutions of Hainan. China Hospitals. (2025) 29:22–6. doi: 10.19660/j.issn.1005-9008.2025.29.05

[22] Xu ZY Xu W Zhang L Wang H Liu J Yang S . Does the "no-filing" policy for cross-province medical services affect the healthcare choices, medical expenses, and insurance funds for urban employees' basic medical insurance?—an empirical study based on a difference-in-differences model. Chinese J Health Policy. (2021) 14:36–41. doi: 10.3969/j.issn.1674-2982.2021.14.005

[23] The State Council of the People's Republic of China. Significance of Integrating the Urban and Rural Residents' Basic Medical Insurance System [EB/OL]. (2016). Available online at: https://www.gov.cn/zhengce/zhengceku/2016-01/12/content_10582.htm (Accessed September 22, 2025).

[24] Longnan Municipal People's Government. The Provincial Coordination of Medical Insurance is Accelerating, What Benefits Will It Bring to the People? [EB/OL]. (2025). Available online at: https://jxln.gov.cn/lnzf/ldxx/202506/e2503a12c68b4c3da6c20124dc21a237.shtml (Accessed December 8, 2025).

[25] Li YH Yan WS Zhang Q Zhao L Sun Y Zhou X . Palliative care, good death, and the social security system: conceptual definitions and theoretical analysis. Insur Stud. (2025) 2:3–13. doi: 10.3969/j.issn.1004-3306.2025.02.001

[26] Hangzhou Net. Promoting the Provincial Overall Planning of Basic Medical Insurance Oriented by Common Prosperity [EB/OL]. (2023). Available online at: https://z.hangzhou.com.cn/2023/bbzl/content/content_8590618.html (Accessed January 15, 2026).

[27] Zeng WF. Provincial-level pooling of basic medical insurance: experiences, dilemmas, and countermeasures. Health Econ Res. (2024) 9:34–7. doi: 10.14055/j.cnki.33-1056/f.2024.09.006

[28] The State Council of the People's Republic of China. Sichuan Introduces Policies to Promote the Provincial Overall Planning of Basic Medical Insurance [EB/OL]. (2023). Available online at: https://www.gov.cn/lianbo/difang/202311/content_6914919.htm (Accessed September 10, 2025).

[29] Yang WX Li JH. Hainan's exploration of the implementation path for provincial-level medical insurance pooling. China Health Insur. (2009) 10:23–5. doi: 10.3969/j.issn.1008-5971.2009.10.010

[30] The State Council of the People's Republic of China. Recent Key Implementation Plan for Medical and Health System Reform (2009–2011) [EB/OL]. (2009). Available online at: https://www.gov.cn/zhengce/content/2009-04/07/content_6239.htm (Accessed October 29, 2025).

[31] The State Council of the People's Republic of China. Plan for Deepening the Reform of the Medical and Health System during the 12th Five-Year Plan Period and its Implementation Plan [EB/OL]. (2012). Available online at: https://www.gov.cn/zwgk/2012-03/21/content_2096671.htm (Accessed November 25, 2025).

[32] Shangluo Municipal Medical Security Bureau. Notice on Printing and Distributing the "14th Five-Year Plan for the Development of Medical Security in Shangluo City" [EB/OL]. (2021). Available online at: https://www.shangluo.gov.cn/PDF/jhgh/145/guanyuyinfashangluoshishisiwuyiliaobaozhangshiyefazhanguihuadetongzhi.pdf (Accessed December 3, 2025).

[33] The State Council of the People's Republic of China. Notice of the General Office of the State Council on Printing and Distributing the "14th Five-Year Plan for Universal Medical Security" [EB/OL]. (2021). Available online at: https://www.gov.cn/zhengce/zhengceku/2021-09/29/content_5639967.htm (Accessed September 17, 2025).

[34] Xinhua Net. Guangxi Continuously Improves the Five-Level Medical Insurance Handling and Management Service System to Open Up the "Last Mile" of Medical Insurance Services [EB/OL]. (2024). Available online at: https://www.gx.xinhuanet.com/20240803/0c69198ed2b146cfb2c0d439c982bc48/c.html (Accessed January 8, 2026).

[35] Nankang District People's Government. Text Interpretation of the Implementation Opinions of the General Office of the People's Government of Jiangxi Province on Promoting the Provincial Coordination and Adjustment of Basic Medical Insurance Funds [EB/OL]. (2024). Available online at: https://www.nkjx.gov.cn/nkqxxgk/c116267/202403/eb575c66bd4b4e05a0e6f815ee8591ce.shtml (Accessed October 5, 2025).

[36] Jiangxi Provincial Medical Security Bureau. Reply Letter to the 0403rd Proposal of the Second Session of the 13th Provincial Committee of the Chinese People's Political Consultative Conference [EB/OL]. (2024). Available online at: https://ybj.jiangxi.gov.cn/jxsylbzj/col/col27601/content/content_1870854477657071616.html (Accessed November 19, 2025).

[37] Nanning Medical Security Bureau. Final Accounts of Medical Insurance Funds at the Municipal Level of Nanning in 2023 [EB/OL]. Available online at: https://ybj.nanning.gov.cn/xxgk_164/czxx/t6164916.html (Accessed September 28, 2025).

[38] Guilin Municipal Medical Security Bureau. Statistical Express on the Development of Medical Security in Guilin in 2023 [EB/OL]. (2024). Available online at: https://ybj.guilin.gov.cn/zfxxgk/fdzdgknr/tjsj/202406/t20240618_2705636.html (Accessed December 12, 2025).

[39] Wuzhou Municipal Finance Bureau. Report of the People's Government of Wuzhou on the Final Accounts of the Municipal Level of Wuzhou in 2023 [EB/OL]. (2024). Available online at: https://czj.wuzhou.gov.cn/xxgk_12036/fdzdgknr_123/czxx_12051/czjs_12053/t18989370.shtml (Accessed January 20, 2026).

[40] Beihai Municipal People's Government of Guangxi. Payment of Medical Insurance Benefits in Beihai in July 2023 [EB/OL]. (2023). Available online at: https://www.beihai.gov.cn/xxgkbm/bhsylbzj/ztzl_6/ylbx_175469/t16970327.shtml (Accessed October 8, 2025).

[41] Fangchenggang Municipal People's Government of Guangxi. Report on the Final Accounts of the Municipal Level of Fangchenggang in 2023 [EB/OL]. (2024). Available online at: https://www.fcgs.gov.cn/zfxxgk/zdlyxxgk/czzj/szfjs/js2023/t19009907.shtml (Accessed November 14, 2025).

[42] Guigang Municipal People's Government of Guangxi. Main Indicators of Basic Medical Insurance in Guigang in 2023 [EB/OL]. (2024). Available online at: https://www.gxgg.gov.cn/xxgk/bmwj/t18665671.shtml (Accessed September 30, 2025).

[43] Yulin Municipal People's Government of Guangxi. Report on the Final Accounts of the Municipal Level of Yulin in 2023 [EB/OL]. (2024). Available online at: https://www.yulin.gov.cn/gkzl/fadingzhudonggongkaineirong/ylsbjyjsgkpt_30014/zfjs/2023n/t19003214.shtml (Accessed December 28, 2025).

[44] Baise Municipal Finance Bureau. Report on the Draft Final Accounts of the Municipal Level of Baise in 2023 [EB/OL]. (2024). Available online at: https://czj.baise.gov.cn/czyjsgkpt/sbjzfyjs_52927/sbjzfjs/t18997118.shtml (Accessed January 5, 2026).

[45] Hechi Municipal People's Government of Guangxi. Quarterly Publicity of Urban Employee Medical Insurance Funds and Urban-Rural Residents Medical Insurance Funds in Hechi in 2023 [EB/OL]. (2024). Available online at: https://www.hechi.gov.cn/tzgg/t19039759.shtml (Accessed October 22, 2025).

